# Income distribution and health: What do we know from Chinese data?

**DOI:** 10.1371/journal.pone.0263008

**Published:** 2022-01-24

**Authors:** Shi Ting, Wenbin Zang, Chen Chen, Dapeng Chen

**Affiliations:** 1 School of Public Administration, Southwestern University of Finance and Economics, Chengdu, Sichuan, China; 2 School of Insurance, Southwestern University of Finance and Economics, Chengdu, Sichuan, China; 3 School of Public Finance & Economics, Shanxi University of Finance and Economics, Taiyuan, Shanxi, China; 4 Department of Economics, Lehigh University, Bethlehem, PA, United States of America; University of South Florida, UNITED STATES

## Abstract

In the last four decades, the problem of income inequality has gradually become one of the most serious social problems in China at both the regional and individual levels. Recently, the central government announced that the main social contradiction is that between people’s growing need for a better life and unbalanced and insufficient economic development. In this study, we analyse the effects of income distribution on individuals’ health using a series of indicators of income distribution and different measures of individuals’ health status. By utilizing data from the China Health and Nutrition Survey (CHNS) from 1989 to 2015, our empirical findings show that self-reported health (SRH), activities of daily living (ADLs), and diabetes mellitus appear to be negatively related to the income share of rich people when average income is equalized among counties, which indicates that individuals’ health will deteriorate as the income share of rich people increases. In addition, our results show that there is an inverted U-shaped relationship between income inequality, as measured by the county-level Gini coefficient, and individuals’ health status. We also find that income inequality affects health through the accessibility of healthcare facilities and public infrastructures and through hazardous health behaviours such as smoking and alcohol use. These findings suggest that reducing income inequality could be an important means of improving the overall health of China’s population.

## 1. Introduction

In the last four decades, the problem of income inequality has gradually become one of the most serious social problems in China. By 2015, China’s Gini coefficient had reached 0.46, and according to international practice, 0.4 is considered as the "warning line" of the income distribution gap between rich people and poor people [1]. Income inequality has an impact on many aspects of social and economic development. In the field of population health, researchers have conducted numerous studies analysing the impact of income inequality on individuals’ health and the possible channels of the effect. There are also many studies that have examined whether the expansion of income inequality worsens health inequality and whether the effects are heterogeneous among different income groups [2–8]. However, the previous findings are mixed.

From the 1970s to the 1990s, multinational macro data were mostly used to analyse the relationship between income distribution and overall population health. The Gini coefficient, the Theil index, and rich people’s share of total income were commonly used to measure income distribution. Regarding health status, researchers often chose life expectancy, the mortality rate, and low birth weight in their analyses [9–11]. The conclusions reached in this period were quite consistent, indicating a negative relationship between income inequality and population health [12–15]. By the mid-1990s, with scepticism regarding the comparability and reliability of cross-border data and the availability of micro level data, researchers started to utilize individual-level data. Consequently, the focus of research shifted from overall population health to individuals’ health and individual health measures, such as self-reported health (SRH), which replaced population health measures [16, 17]. After controlling for family and personal characteristics and the mean income of regions, most empirical studies found a negative relationship between the Gini coefficient and SRH [18, 19]. Kawachi and Kennedy (1999) [20, 21] showed that the widening gap between rich people and poor people caused serious social polarization, destroyed the social relationship between people, reduced social trust and social cohesion, and affected the health of members of society. Spencer (2004) [22] reached similar conclusions and pointed out that the wide income gap between rich people and poor people made low-income individuals feel depressed, which could exert significant psychological pressure and lead to negative emotions. When people live under such conditions, their risk of suffering from various chronic diseases, such as cardiovascular disease and depression, increases. In contrast, by using American data, Mellor and Milyo (2001) [17] found that there was a significant relationship between the Gini coefficient and SRH when considering only income level and the Gini coefficient, however, after personal characteristics were controlled for in the regression equations, the impact of income inequality on SRH was no longer significant.

Regarding studies using Chinese data, the findings are also mixed. Using two waves of data (1997 and 2000) from the China Health and Nutrition Survey (CHNS), Feng and Yu (2007) [23] found an inverted U-shaped relationship between county-level income inequality and SRH. Li and Zhu (2008) [16] found a significant association between community-level income inequality, as measured by the Gini coefficient, and SRH. In contrast, Chen and Meltzer (2008) [24] showed that there was no relationship between county-level income inequality, as measured by the Gini coefficient, and individuals’ health, as measured by obesity and hypertension, in urban China. Bakkeli (2016) [25] found that the Gini coefficient did not have a significant impact on individuals’ risks of having health problems, as measured by blood pressure and mid-arm muscle circumference (MAMC). Additionally, their results were robust when using the Theil indexes to measure income distribution.

The possible mechanisms of the effects of income inequality on individuals’ health are as follows. First, income inequality could be a major source of social distrust and stress, which could have a direct influence on people engaging in behaviours that are harmful to their health (e.g., smoking and drinking) [26]. Second, when average income is equalized among communities, the larger the income inequality in a community is, the greater the likelihood that the low-income population will suffer from insufficient healthcare due to financial constraints. Third, income inequality will affect the accessibility of healthcare facilities and public infrastructures for poor people who are more likely to live in rural areas where the supply of public goods is usually insufficient due to unbalanced economic development [19, 27].

This study utilizes a longitudinal dataset set to analyse the relationship between income distribution and individuals’ health in China. We make several contributions to the existing literature. First, we measure the income distribution in multiple dimensions, including the share of county’s total income held by the richest 5% of the population, the Gini coefficient, and the Theil index. Different measures have different emphases, and by including multi-dimensional measures of income inequality, we are able to analyse the impact of income distribution on health more comprehensively. Second, most of the previous literature adopted only one subjective health measure, SRH, which may not fully reflect individuals’ health status. In addition to SRH, this study analyses several objective health indexes, including body mass index (BMI), activities of daily living (ADLs), and diabetes. Third, previous studies have mainly focused on the impact of income distribution on health status, and very few have expanded the discussion to the channels of the impact of income distribution. This study attempts to analyse whether income inequality affects health through the accessibility of healthcare facilities and public infrastructures and through hazardous health behaviours such as smoking and alcohol consumption. Lastly, we utilize a balanced panel to alleviate the problem of endogeneity and examine the dynamic effect of income distribution on individuals’ health. In short, our empirical findings show that SRH, ADLs and diabetes are negatively related to the income share of rich people when average income is equalized among counties. Additionally, there is an inverted U-shaped relationship between the county-level Gini coefficient and individuals’ health. Income inequality may also affect health through the accessibility of healthcare facilities and public infrastructures and through hazardous health behaviours.

## 2. Data and methods

### 2.1. Data source

The sample used in this study is drawn from ten waves (1989–2015) of the CHNS. The CHNS was designed to investigate Chinese residents’ health and nutrition status and how this status is affected by the social and economic transformation of Chinese society. Based on a multi-stage stratified random cluster process, the survey draws 7,200 households with over 30,000 individuals in both urban and rural areas from 15 provinces and municipal cities representing most parts of China’s territory. We further restrict our sample to individuals who are at least 16 years old with full-scale information on their health status and other personal characteristics. As we need to construct income inequality indexes, families with zero income or less are also excluded. Finally, we obtain two sub-samples for analysis: pooled cross-sectional data consisting of 78,235 observations in total and a balanced panel of 121 observations from each wave.

### 2.2. Dependent variables

Our dependent variables are various measures of individuals’ health conditions, including BMI, SRH, ADLs, and diabetes, which are transformed into binary variables. Specifically, BMI is a widely used measurement for obesity and health status. The standard value ranges between 18.5 and 23.9, and a higher or lower value outside of that range indicates an unhealthy status. In this study, we regard a person as healthy if his or her BMI falls between 18.5 and 23.9 and unhealthy otherwise. In CHNS data, SRH includes five different categories (“very good”, “good”, “fair”, “bad”, and “very bad”). We use a dummy variable that takes the value of 1 if the survey respondent reports a good or very good health condition and 0 otherwise. ADLs are a series of basic activities necessary for a person’s independent living at home or in the community [28, 29]. They mainly consist of personal hygiene, dressing, eating, maintaining continence, and mobility. We applied a binary variable that indicates whether the respondents could complete all of these basic activities. Finally, diabetes indicates whether a respondent was diagnosed with the disease.

### 2.3. Independent variables

The key independent variables are the county-level share of rich people, the Gini coefficient, the Theil-index, and the percentile expenditure ratio (P90/P10). The county-level share of rich people is the share of the county’s total income received by a certain percentage of the population, usually 5%, 10%, etc. It could specify the income share of people with either the highest or lowest income. The Gini coefficient is an index (between 0 and 1) used to evaluate the degree of equality of a distribution based on the Lorentz curve. The more equal the income distribution is, the smaller the arc of the Lorentz curve and the smaller the Gini coefficient. As a measure of inequality, the Theil index has good decomposability, which means that when a sample is divided into multiple groups, the Theil index can measure the contribution of intra-group and inter-group inequality to the total inequality. P90/P10 represents the proportion of income in the 90th percentile to that in the 10th percentile. We use different indexes to reflect the income distribution within county-level administrative regions, which include both counties in rural areas and districts in urban areas. Using county-level administrative regions as the unit of analysis is reasonable because counties are a basic government branch in the fiscal system and are responsible for providing of most healthcare services and other public infrastructures.

Table 1 lists all individual-level variables, including income proxied by per capita family income, length of education, age, gender, ethnicity, marital status, occupation, hukou (urban or rural resident), and health insurance status [30]. Moreover, we control for several public infrastructure covariates, such as accessibility to tap water and the availability of indoor toilets, and other variables that reflect the supply of healthcare, including the number of medical institutions and the distance to the closest medical institution.

**Table 1:** Summary statistics.

Variables	Obs.	Mean	Std.dev	Min	Max
**Health Variables**					
BMI (Normal = 1)	78235	.598	.49	0	1
SRH (Very good/good = 1)	29588	.659	.474	0	1
ADL (No difficulty = 1)	7950	.775	.417	0	1
Diabetes (No = 1)	46503	.978	.145	0	1
**Health Behaviors**					
Daily Smoking	19068	15.81	8.613	1	40
Drinking Frequency	22780	3.258	1.421	0	5
**Income and Income Distribution**					
Per Capita County Income (Ln)	78235	8.88	.996	1.193	13.94
Per Capita Family Income (Ln)	78235	8.825	1.298	.361	15.326
Gini Coefficient	78235	.463	.035	.323	.544
Rich_share5	78235	12.119%	7.227	2.937	64.358
Rich_share10	78235	19.509%	9.951	3.981	75.999
Rich_share20	78235	35,710%	10.489	7.025	86.391
Theil Index	78235	.427	.091	.208	.691
P90/P10	78235	13.096	4.248	3.699	26.168
**Other Variables**					
Age	78235	45.103	15.384	16	100
Marriage (Yes = 1)	78235	.831	.375	0	1
Gender (Male = 1)	78235	.512	.5	0	1
Education	78235	7.578	4.244	0	18
Insurance (Yes = 1)	78235	.889	.314	0	1
Occupation	78235	3.822	1.509	1	6
Hukou (Rural = 1)	78235	.654	.476	0	1
Tap water (Yes = 1)	78235	.74	.439	0	1
Indoor toilet (Yes = 1)	78235	.437	.496	0	1
Family size	78235	3.924	1.585	1	15
Number of Medical Institutions	78235	1.277	.548	1	8
Distance (Minutes)	78235	14.546	13.844	4	90

### 2.4. Empirical models

This study applies a probit model as our dependent variables are all binary. In Eq (1), we first use the share of rich people as the measure of income distribution to examine the influence of income inequality on individuals’ health:

$$Probit(Y_{ict}=1)=\Phi (\beta_{0}+\beta_{1}{Lnrich\_share}_{ct}+\gamma X_{ict}+\mu_{ict})$$ (1)

where subscripts *i*, *c*, and *t* denote that individual *i* from county *c* was interviewed in year *t*. The dependent variable is a binary variable indicating whether person *i* was in a good health condition or not. *β*_1_ is the coefficient of interest, which shows the association of individuals’ health with the share of county income received by rich people after controlling for the average income of the county and other covariates. We use different shares (5%, 10% and 20%) t of rich people to estimate the impacts of income inequality in a county. *X* is a vector of covariates including per capita county income and the other aforementioned covariates. *μ* is the independently and identically distributed error term.

In addition, we use the county-level Gini coefficient/Theil-index as measures of income distribution, and an interaction term between per capita family income and the inequality indexes is added. This paper estimates the Gini coefficient at the county level. At present, China’s counties are not only the basic unit in the financial system but also the basic scope of farmers’ economic activities. County governments are fully responsible for public health and basic health services, especially in rural areas [23]. Subramanian and Kawachi (2004) [19] believe that the US state-level governments are responsible for implementing policy with regard to residents’ public health and education investment as well as other income redistribution policies. The use of state-level income gap indicators has a significant impact on health, while at a lower level, the role of the income gap is weak. Combined with the situation of China, the scope of social communication and economic activities has exceeded the scope of communities and towns; thus, it is necessary to investigate the income gap and its influence mechanism at a higher level. Therefore, it is reasonable to estimate the income gap by county-level units in China. Specifically, the estimation equation is as follows:

$$Probit(Y_{ict}=1)=\Phi (\beta_{0}+\beta_{1}{Gini}_{ct}+\beta_{2}{Gini}_{ct}*{Lnincome}_{ct}+\gamma X_{ict}+\mu_{ict})$$ (2)

where *Y*_*ict*_ and *X*_*ict*_ are similarly defined as in Eq (1). Per capita family income is used as the income variable in this paper. It primarily reflects the fact that an individual’s health may depend more on family resources than on an individual’s salary level because family members often make decisions based on the whole family’s economic circumstances. The family is both a production unit and a decision-making unit; thus, per capita family income can more accurately reflect the effect of income and the income gap on health than the income of the individual as a member of the labour force. In addition, the equalization of total family income can better capture the utilization of family resources by all family members. *Gini*_*ct*_ is the Gini coefficient of county *c* in year *t*, and *β*_1_ is the key coefficient of interest. *Gini*_*ct*_ is replaced by *Theil*_*ct*_ when the Theil-index is used as the measure of income distribution. The interaction term of the Gini coefficient or Theil-index and per capita family income can help us explore the heterogeneous effects of income inequality on the health of people with different income levels.

## 3. Results

### 3.1. The share of rich people and individuals’ health

The income share of the richest 5% of the population (rich_share5) was calculated by dividing the total income of the richest 5% of the population by total income. Table 2 reports the regression estimates. We find that the income share of the richest 5% of the population has a significant negative effect on all health status measures except for BMI after controlling for the average income of the county and individual and family characteristics. Specifically, for every 1% increase in the share of rich people (rich_share5), the probability of being in very good health/good health decreases by 4.81%, the probability of having no difficulties with ADLs declines by 6.16%, and the probability of being diagnosed with diabetes increases by 5.16%. The findings demonstrate that individuals’ health will worsen as the income share of rich people increases. The same results are not found when BMI is used as the measure of health status. BMI is a diagnostic tool for obesity that can predict a person’s health conditions; however, the cut-off points (lower than 18.5 or higher than 23.9) are ambiguous, and thus, it is not a well-recognized indicator of health [31]. Therefore, it is not surprising that our estimates show that present income inequality and BMI are not related. Regarding the control variables, the higher the per capita family income is, the better the SRH and ADLs, the worse the BMI is, the higher the risk of diabetes. County average income has a significant positive effect on all health status measures except for diabetes. Individuals’ health status decreases with age, and men have a better health status than women. Unmarried people have a better BMI and lower rates of diabetes. The greater the number of years of education an individual has, the better his or her SRH, probably because he or she is more health conscious. Insurance status has no significant effect on individuals’ health status. We find that having more family members is beneficial to the health of the individual, especially if elderly individuals have difficulty moving and family members can provide necessary help. In addition, rural residents have a better BMI and a lower risk of diabetes, which is related to their diet structure and eating habits.

**Table 2:** The effects of rich share (5%) on individual health.

Variables	BMI	SRH	ADL	Diabetes
	(1)	(2)	(3)	(4)
Lnrich_share5	0.0275	-0.0481**	-0.0616***	-0.0516**
	(0.0200)	(0.0197)	(0.0154)	(0.0212)
Ln(Per Capita Family Income)	-0.0600***	0.0354*	0.0703**	-0.0670**
	(0.0110)	(0.0182)	(0.0290)	(0.0332)
Ln(Per Capita County Income)	0.0119*	0.0614***	0.0345*	-0.0367
	(0.00620)	(0.0106)	(0.0187)	(0.0283)
Age	-0.00617***	-0.0235***	-0.0569***	-0.0268***
	(0.000720)	(0.00107)	(0.00318)	(0.00162)
Gender	0.0470**	0.163***	0.239***	0.0393
	(0.0187)	(0.0187)	(0.0401)	(0.0437)
Married	-0.0858***	0.0300	-0.0489	-0.0921*
	(0.0195)	(0.0295)	(0.0528)	(0.0477)
Education	0.00235	0.0103***	0.0143**	-0.0109*
	(0.00278)	(0.00374)	(0.00566)	(0.00586)
Insurance	0.0401	0.0618	-0.117**	0.0933
	(0.0255)	(0.0376)	(0.0581)	(0.0579)
Family size	0.0199***	-0.00111	0.0363***	0.0381**
	(0.00570)	(0.0115)	(0.0131)	(0.0158)
Hukou	0.101***	0.0308	0.0596	0.184***
	(0.0369)	(0.0480)	(0.0675)	(0.0664)
Year	Yes	Yes	Yes	Yes
Obs	78,235	29,588	7,950	46,503
R^2^	0.206	0.272	0.281	0.296

Note: Standard deviations in parentheses

* p < 0.1

** p < 0.05

*** p < 0.01.

We also estimate the relationship between the share of the county’s total income received by the richest 10% and 20% of the population and the indicators of individuals’ health (Tables 3 and 4). The findings are highly consistent with those reported in Table 2. Again, individuals’ health is negatively affected by income inequality when SRH, ADLs, and diabetes are used as health measures. The coefficient on income inequality remains nonsignificant when health is measured by BMI. The sign and significance of the control variables are also basically consistent with those shown in Table 2.

**Table 3:** The effects of rich share (10%) on individual health.

Variables	BMI	SRHS	ADL	Diabetes
	(1)	(2)	(3)	(4)
Lnrich_share10	0.0159	-0.0268***	-0.0467***	-0.0895**
	(0.0179)	(0.00160)	(0.0133)	(0.0440)
Ln(Per Capita Family Income)	-0.0602***	0.0354*	0.0701**	-0.0671**
	(0.0110)	(0.0182)	(0.0290)	(0.0332)
Ln(Per Capita County Income)	0.0119*	0.0613***	0.0337*	-0.0362
	(0.00620)	(0.0106)	(0.0187)	(0.0281)
Age	-0.00619***	-0.0235***	-0.0570***	-0.0268***
	(0.000719)	(0.00107)	(0.00319)	(0.00160)
Gender	0.0470**	0.163***	0.239***	0.0381
	(0.0187)	(0.0187)	(0.0401)	(0.0435)
Married	-0.0852***	0.0293	-0.0497	-0.0904*
	(0.0195)	(0.0296)	(0.0528)	(0.0473)
Education	0.00233	0.0103***	0.0144**	-0.0107*
	(0.00277)	(0.00375)	(0.00560)	(0.00584)
Insurance	0.0419	0.0590	-0.121**	0.0959*
	(0.0257)	(0.0373)	(0.0584)	(0.0578)
Family size	0.0198***	-0.00115	0.0361***	0.0383**
	(0.00571)	(0.0115)	(0.0131)	(0.0158)
Hukou	0.0998***	0.0324	0.0605	0.184***
	(0.0369)	(0.0478)	(0.0675)	(0.0662)
Year	Yes	Yes	Yes	Yes
Obs	78,235	29,588	7,950	46,503
R^2^	0.214	0.301	0.321	0.342

Note: Standard deviations in parentheses

* p < 0.1

** p < 0.05

*** p < 0.01.

**Table 4:** The effects of rich share (20%) on individual health.

Variables	BMI	SRHS	ADL	Diabetes
	(1)	(2)	(3)	(4)
Lnrich_share20	0.0267	-0.0294**	-0.0461**	-0.0672*
	(0.0290)	(0.0130)	(0.0188)	(0.0353)
Ln(Per Capita Family Income)	-0.0602***	0.0349*	0.0706**	-0.0675**
	(0.0110)	(0.0182)	(0.0290)	(0.0334)
Ln(Per Capita County Income)	0.0121*	0.0607***	0.0338*	-0.0358
	(0.00620)	(0.0107)	(0.0188)	(0.0282)
Age	-0.00618***	-0.0235***	-0.0570***	-0.0268***
	(0.000719)	(0.00107)	(0.00320)	(0.00160)
Gender	0.0469**	0.163***	0.239***	0.0385
	(0.0187)	(0.0187)	(0.0402)	(0.0436)
Married	-0.0853***	0.0291	-0.0503	-0.0901*
	(0.0195)	(0.0297)	(0.0529)	(0.0473)
Education	0.00235	0.0102***	0.0144**	-0.0108*
	(0.00277)	(0.00375)	(0.00563)	(0.00583)
Insurance	0.0415	0.0614	-0.120**	0.0954*
	(0.0256)	(0.0373)	(0.0587)	(0.0576)
Family size	0.0197***	-0.000600	0.0364***	0.0384**
	(0.00572)	(0.0114)	(0.0131)	(0.0158)
Hukou	0.0999***	0.0315	0.0594	0.183***
	(0.0370)	(0.0479)	(0.0675)	(0.0665)
Year	Yes	Yes	Yes	Yes
Obs	78,235	29,588	7,950	46,503
R^2^	0.215	0.291	0.311	0.313

Note: Standard deviations in parentheses

* p < 0.1

** p < 0.05

*** p < 0.01.

### 3.2. Gini coefficient and individuals’ health

In this section, to measure income distribution, we use the Gini coefficient, which is a commonly used index reflecting the degree of income inequality between rich people and poor people [32]. Tables 5 and 6 report the regression results. We first estimate Eq (2) by including only the Gini coefficient, its squared term, and other covariates (Model 1). We then add an interaction term between the Gini coefficient and income level to test the heterogeneous effects of income inequality on populations with different income levels (Model 2). The results indicate that there is an inverted-U shaped correlation between the Gini coefficient and SRH, ADLs, and diabetes, meaning that the individuals’ health status will first improve with an increase in income inequality; however, health conditions will deteriorate significantly once a threshold is exceeded. The Gini coefficient thresholds for SRH, ADLs, and Diabetes are 0.456, 0.442, and 0.425, respectively. According to the Bureau of National Statistics, the Gini coefficient in China in 2015 was 0.462, exceeding all three threshold estimates. Similarly, Feng and Yu (2007) [23] used two waves of CHNS data (1997 and 2000) to show that there was an inverted U-shaped relationship between the Gini coefficient and SRH, and the threshold point was 0.35. Using 1993 CHNS data, Li and Zhu (2008) [16] found that the threshold of the Gini coefficient was 0.40. Again, BMI is not significantly affected by the Gini coefficient, which similar to the previous findings using the share of rich people as the measure of income inequality.

**Table 5:** The effects of Gini coefficient on BMI and SRH.

Variables	BMI		SRH	
	(1)	(2)	(1)	(2)
Gini	-0.287	0.850	17.86***	21.22***
	(7.086)	(7.352)	(3.572)	(4.422)
Gini Squared	-1.045	-1.069	-19.56***	-19.85***
	(7.132)	(7.131)	(4.091)	(4.292)
Ln(Per Capita Family Income)	-0.00802*	-0.00799*	0.0196***	0.0195***
	(0.00465)	(0.00465)	(0.00297)	(0.00297)
Gini* Ln(Per Capita Family Income)		-0.132		0.155*
		(0.229)		(0.086)
Age	-0.0154***	-0.0154***	-0.00788***	-0.00788***
	(0.000616)	(0.000616)	(0.000228)	(0.000228)
Gender	0.0647***	0.0647***	0.0527***	0.0527***
	(0.0105)	(0.0105)	(0.00608)	(0.00608)
Married	-0.00873	-0.00861	0.00693	0.00696
	(0.0123)	(0.0123)	(0.00789)	(0.00789)
Education	0.00358***	0.00358***	0.00359***	0.00358***
	(0.00128)	(0.00128)	(0.000881)	(0.000881)
Insurance	-0.0299**	-0.0301**	0.0155*	0.0153*
	(0.0120)	(0.0120)	(0.00810)	(0.00810)
Family size	0.00851***	0.00842***	-0.000145	-0.000182
	(0.00272)	(0.00273)	(0.00202)	(0.00202)
Hukou	0.0140	0.0141	0.00982	0.00980
	(0.0125)	(0.0125)	(0.00785)	(0.00785)
Year	Yes	Yes	Yes	Yes
Obs	78,235	78,235	29,588	29,588
R^2^	0.193	0.249	0.253	0.269

Note: Standard deviations in parentheses

* p < 0.1

** p < 0.05

*** p < 0.01.

**Table 6:** The effects of Gini coefficient on ADL and diabetes.

Variables	ADL		Diabetes	
	(1)	(2)	(1)	(2)
Gini	2.103**	2.424**	0.0493***	0.165**
	(0.943)	(1.064)	(0.009)	(0.073)
Gini Squared	-2.378**	-2.279**	-0.058***	-0.264
	(1.086)	(1.109)	(0.01)	(0.909)
Ln(Per Capita Family Income)	0.00400**	0.00399**	-0.00114	-0.00107
	(0.00169)	(0.00169)	(0.000892)	(0.000893)
Gini* Ln(Per Capita Family Income)		0.175**		0.0562**
		(0.073)		(0.023)
Age	-0.00271***	-0.00272***	-0.00123***	-0.00125***
	(0.000186)	(0.000186)	(7.93e-05)	(8.12e-05)
Gender	0.00653	0.00655	0.00101	0.00100
	(0.00531)	(0.00531)	(0.00173)	(0.00175)
Married	-0.0198***	-0.0197***	-0.00546**	-0.00544**
	(0.00484)	(0.00484)	(0.00221)	(0.00221)
Education	-0.000551	-0.000555	-0.000386*	-0.000404*
	(0.000640)	(0.000640)	(0.000213)	(0.000214)
Insurance	0.0151***	0.0150***	0.00102	0.000595
	(0.00529)	(0.00530)	(0.00217)	(0.00218)
Family size	0.00262**	0.00261**	0.00143***	0.00144***
	(0.00120)	(0.00120)	(0.000531)	(0.000534)
Hukou	0.0392***	0.0392***	0.0108***	0.0111***
	(0.00602)	(0.00602)	(0.00195)	(0.00202)
Year	Yes	Yes	Yes	Yes
Obs	7,950	7,950	46,503	46,503
R^2^	0.365	0.367	0.380	0.384

Note: Standard deviations in parentheses

* p < 0.1

** p < 0.05

*** p < 0.01.

In Model 2, we include the interaction term between the Gini coefficient and income level to check the heterogeneous effects of income inequality. The estimates in Tables 5 and 6 show that the coefficients on the interaction terms are positive when SRH, ADLs, and diabetes are used as the health measures, meaning that an increase in income inequality will amplify the effect of income level on individuals’ health. In other words, the expansion of income inequality has caused the health conditions of poor people to deteriorate more other the study people than their rich counterparts, further widening the health gap between rich people and poor people. Regarding the control variables, the results are consistent with those shown in Table 2.

### 3.3. Possible channels

Why might increasing income inequality be correlated with worse health status? In this section, we propose several possible channels through which income distribution affects individuals’ health status, including the accessibility of health care facilities and public infrastructures as well as smoking and alcohol use.

#### 3.3.1. The accessibility of health facilities and public infrastructures

A very high level of income possessed by rich people may indicate uneven regional development, and poor people living in underdeveloped areas are likely to have limited access to healthcare facilities and public infrastructures. A large number of studies have confirmed the impact of the accessibility of healthcare facilities and public infrastructures on health conditions [33]. In this study, we use the number of medical institutions in a county per capita and the distance to the closest medical institution to measure the accessibility of healthcare facilities. Additionally, we use tap water and indoor toilets to proxy for the availability of public infrastructures. From the regression results in Tables 7 and 8, we find that the impact of the accessibility of healthcare facilities and public infrastructures on health status is mostly positive and significant. For example, the higher the number of hospitals per capita in a county is, the better the health status of residents will be. More importantly, the absolute values of the coefficients on income inequality (Lnrich_share5) are smaller than those reported in Table 2, which means that income distribution affects individuals’ health status through the accessibility of healthcare facilities and public infrastructure investment.

**Table 7:** Possible channels of influence of income inequality.

Variables	BMI		SRH	
	(1)	(2)	(1)	(2)
Lnrich_share5	0.0275	0.0278	-0.0481**	-0.0410**
	(0.0200)	(0.0200)	(0.0229)	(0.0178)
Toilet		0.0669*		0.161***
		(0.0393)		(0.0186)
Water		0.0476*		0.0107***
		(0.0273)		(0.00368)
Num_hospitals(per capita)		0.0143***		0.0527*
		(0.00487)		(0.031)
Distance		-0.000563		-0.00167**
		(0.000602)		(0.000806)
Control X	Yes	Yes	Yes	Yes
Year	Yes	Yes	Yes	Yes
Obs	78,235	78,235	29,588	29,588
R^2^	0.091	0.167	0.157	0.201

Note: Standard deviations in parentheses

* p < 0.1

** p < 0.05

*** p < 0.01.

**Table 8:** Possible channels of influence of income inequality.

Variables	ADL		Diabetes	
	(1)	(2)	(1)	(2)
Lnrich_share5	-0.0616***	-0.0521***	-0.0516**	-0.0264***
	(0.0154)	(0.00318)	(0.0212)	(0.00157)
Toilet		0.0155		0.0407**
		(0.0578)		(0.0159)
Water		0.0143**		0.156**
		(0.00574)		(0.0699)
Num_hospitals(per capita)		0.0385***		0.0279**
		(0.0133)		(0.0126)
Distance		-0.0837		(0.00174)
		(0.0629)		(0.000962)
Control X	Yes	Yes	Yes	Yes
Year	Yes	Yes	Yes	Yes
Obs	7,950	7,950	46,503	46,503
R^2^	0.169	0.223	0.276	0.295

Note: Standard deviations in parentheses

* p < 0.1

** p < 0.05

*** p < 0.01.

Next, we directly estimate whether income inequality affects the accessibility of healthcare and public infrastructures (Table 9). The coefficients on the share of rich people and the Gini coefficient are significantly negative, which means that a greater income gap is associated with fewer medical institutions and public infrastructures. These findings support our hypothesis that income inequality affects individual health through the accessibility of healthcare and public infrastructure. Increasing the supply of medical facilities and public infrastructures may help alleviate the adverse impact of the income gap on individual health.

**Table 9:** The effects of income inequality on the provision of health facilities and public infrastructures.

Variables	Num_Hospitals(per capita)		Water		Toilet	
	(1)	(2)	(1)	(2)	(1)	(2)
Lnrich_share5	-0.00588***		-0.0197*		-0.187***	
	(0.00165)		(0.0108)		(0.0223)	
Gini		-0.0225***		-4.939***		-6.981***
		(0.0045)		(0.545)		(0.599)
Control X	Yes	Yes	Yes	Yes	Yes	Yes
Year	Yes	Yes	Yes	Yes	Yes	Yes
Obs	78,235	78,235	78,235	78,235	78,235	78,235
R^2^	0.373	0.385	0.157	0.201	0.091	0.167

Note: Standard deviations in parentheses

* p < 0.1

** p < 0.05

*** p < 0.01.

#### 3.3.2. Hazardous health behaviours

Foster et al. (2018) [34] found that low-income people with lower social positions are more likely to be burdened with more pressure and to engage in unhealthy behaviours. The previous literature has shown that income disparity affects personal health by increasing hazardous health behaviours. For example, Ramos et al. (2017) [35] found that self-reported violent victimization is associated with an increased chance of engagement in hazardous health behaviours in all Brazilian state capitals, especially in areas with serious income inequality. We verify this potential channel by analysing whether income equality affects the probability of individuals engaging in hazardous health behaviours, such as smoking and alcohol use. Table 10 shows that there is a significant and positive correlation between income inequality and the amount of daily smoking and the frequency of drinking, and the results indicate that greater income inequality results in people engaging in hazardous health behaviours.

**Table 10:** Income inequality and health hazardous behaviors.

Variables	Daily Smoking		Drinking Frequency	
	(1)	(2)	(1)	(2)
Lnrich_share5	0.239*		0.0337*	
	(0.134)		(0.0187)	
Gini		7.256***		1.891***
		(2.549)		(0.353)
Ln(Per Capita Family Income)	0.318***	0.295***	0.0337*	0.0322**
	(0.103)	(0.108)	(0.0187)	(0.0151)
Year	Yes	Yes	Yes	Yes
Obs	19,068	19,068	22,780	22,780
R^2^	0.253	0.231	0.157	0.164

Note: Standard deviations in parentheses

* p < 0.1

** p < 0.05

*** p < 0.01.

### 3.4. Robustness check

#### 3.4.1. Theil index and the percentile expenditure ratio (P90/P10)

When measuring income distribution, different income inequality indexes have different emphases. In this section, we use two different measures of income distribution to check for the robustness of our earlier findings. We re-estimate Eqs (1) and (2) by replacing the share of rich people and the Gini coefficient with the Theil index and P90/P10. The Theil index uses the concept of entropy in information theory to calculate income inequality. The advantage of the Theil index is that it can be decomposed to measure the contribution of both intra-group and inter-group income inequality to total income inequality. P90/P10 represents the proportion of income in the 90th percentile to that in the 10th percentile.

The regression results in Tables 11 and 12 show that after replacing the share of rich people and the Gini coefficient with the Theil index and P90/P10, the coefficients of interest barely change in both sign and level of significance. We also find an inverted U-shaped relationship between the Theil index and P90/P10, on the one hand, and SRH, ADLs, and diabetes, on the other hand. The average Theil index and P90/P10 are on the right side of the inverted U-shaped curve, which also indicates that China’s current income distribution has a negative impact on individuals’ health, which is similar to the findings reported in section 3.1. In addition, the coefficients on the interaction term between the Theil index and P90/P10 and income level are significantly positive, which is also consistent with our earlier findings.

**Table 11:** Different index—Theil index.

**Variables**	**BMI**		**SRH**	
	**(1)**	**(2)**	**(1)**	**(2)**
Theil	0.868	-0.854	1.07***	2.72***
	(0.72)	(1.5)	(0.214)	(0.544)
Theil Squared	-1.150	-1.357	-1.41***	-2.98***
	(1.2105)	(1.09)	(0.082)	(0.599)
Theil*Lnincome		0.0191		0.013*
		(0.014)		(0.007)
Year	YES	YES	YES	YES
Obs	78235	78235	7950	7950
R^2^	0.131	0.176	0.191	0.214
**Variables**	**ADL**		**Diabetes**	
	**(1)**	**(2)**	**(1)**	**(2)**
Theil	7.288**	5.579*	7.303***	9.28***
	(2.986)	(3.381)	(1.46)	(1.856)
Theil Squared	-7.685**	-6.76**	-7.937***	-6.17***
	(3.341)	(2.939)	(1.5874)	(1.234)
Theil*Lnincome		0.303**		0.23*
		(0.1212)		(0.139)
Year	YES	YES	YES	YES
Obs	60692	60692	46503	46503
R^2^	0.287	0.291	0.271	0.279

Note: Standard deviations in parentheses

* p < 0.1

** p < 0.05

*** p < 0.01.

**Table 12:** Different index—P90/P10.

**Variables**	**BMI**		**SRH**	
	**(1)**	**(2)**	**(1)**	**(2)**
P90/P10	0.0007	0.0343***	0.0596***	0.0561***
	(0.001)	(0.007)	(0.009)	(0.0112)
P90/P10 Squared	-0.0002	-0.0003**	-0.00653***	-0.00144***
	(0.0002)	(0.0001)	(0.0013)	(0.0002)
P90/P10 * Lnincome		0.00326**		0.0232**
		(0.0015)		(0.009)
Year	YES	YES	YES	YES
Obs	78235	78235	78235	78235
R^2^	0.119	0.120	0.162	0.167
**Variables**	**ADL**		**Diabetes**	
	**(1)**	**(2)**	**(1)**	**(2)**
P90/P10	0.265***	0.56***	0.0719***	0.105*
	(0.0453)	(0.112)	(0.0263)	(0.0570)
P90/P10 Squared	-0.0369***	-0.075***	-0.0081*	-0.0018*
	(0.0073)	(0.013)	(0.0049)	(0.0009)
P90/P10 * Lnincome		0.0754**		0.00208
		(0.0314)		(0.0023)
Year	YES	YES	YES	YES
Obs	46785	46785	7950	7950
R^2^	0.211	0.211	0.123	0.147

Note: Standard deviations in parentheses

* p < 0.1

** p < 0.05

*** p < 0.01.

#### 3.4.2. Simultaneous causality

In this topic, the most common problem is simultaneous causality, which directly affects the reliability and accuracy of our estimates. On the one hand, income inequality will have an effect on individuals’ health behaviours and, thus, their health outcomes; on the other hand, health status influences individuals’ labour force participation and, thus, their personal income. However, current health status does not affect either income inequality or income in the past. Therefore, we substitute the current Gini coefficient and share of rich people with their lagged terms. The results in Table 13 show that the inverted U-shaped relationship between the lagged Gini coefficient and health outcomes still exists when SRH, ADLs, and diabetes are used as health measures. The coefficients of the lagged share of rich people are significantly negative, which is consistent with the results shown in Tables 2–4.

**Table 13:** The effects of lagged income inequality on health.

**Variables**	**BMI**		**SRHS**	
	**(1)**	**(2)**	**(1)**	**(2)**
Lagged Gini	4.419		3.989**	
	(3.506)		(1.628)	
Lagged Gini Squared	-4.710		-4.364**	
			(1.781)	
	(3.870)			
Lagged Lnrich_share5		-0.0189		-0.0540***
		(0.0228)		(0.0200)
Year	YES	YES	YES	YES
Obs	52,739	52,739	21,657	21,657
R^2^	0.231	0.252	0.321	0.353
**Variables**	**ADL**		**Diabetes**	
	**(1)**	**(2)**	**(1)**	**(2)**
Lagged Gini	2.104***		1.632**	
	(0.420)		(0.666)	
Lagged Gini Squared	-2.897**		-3.97*	
	(1.176)		(2.37)	
Lagged Lnrich_share5		-0.0525**		-0.0934**
		(0.0214)		(0.0381)
Year	YES	YES	YES	YES
Obs	5,938	5,938	32,684	32,684
R^2^	0.432	0.443	0.472	0.473

Note: Standard deviations in parentheses

* p < 0.1

** p < 0.05

*** p < 0.01.

#### 3.4.3. Balanced panel data

In this part, we utilize a balanced panel dataset from the ten waves of data from 1989 to 2015, and the sample includes 121 observations from each wave. The balanced panel can alleviate the problem of endogeneity and examine the dynamic effect of income distribution on individual health status. Table 14 presents the regression results. Although an inverted U-shaped relationship between income inequality and health status still exists, the inflection points when SRH, ADLs, and diabetes are used as health measures are 0.274, 0.387, and 0.256, respectively, which are lower than those found in Tables 5 and 6. The coefficients on the share of rich people are still negative and significant. In general, the estimates using balanced panel data are consistent with those reported in previous sections.

**Table 14:** Estimates from balanced panel data.

**Variables**	**BMI**		**SRH**	
	**(1)**	**(2)**	**(1)**	**(2)**
Gini	7.47		3.11**	
			(1.352)	
	(8.48)			
Gini Squared	-5.242		-5.68**	
	(8.19)		(2.469)	
Lnrich_share5		-0.0722		-0.115**
				(0.0469)
		(0.135)		
Year	YES	YES	YES	YES
Obs	1,210	1,210	1,210	1,210
R^2^	0.322	0.324	0.366	0.366
**Variables**	**ADL**		**Diabetes**	
	**(1)**	**(2)**	**(1)**	**(2)**
Gini	0.96**		0.651*	
	(0.417)		(0.394)	
Gini Squared	-1.24**		-1.270*	
	(0.539)		(0.769)	
Lnrich_share5		-0.099***		-0.025*
		(0.0174)		(0.151)
Year	YES	YES	YES	YES
Obs	710	710	920	920
R^2^	0.353	0.356	0.358	0.358

Note: Standard deviations in parentheses

* p < 0.1

** p < 0.05

*** p < 0.01.

## 4. Discussion and conclusion

This study utilizes CHNS data to estimate the impact of income distribution on individuals’ health, and it discusses the possible channels of the effect. The main findings are as follows. First, increasing income inequality has a negative impact on SRH, ADLs, and diabetes when income inequality is measured by the share of rich people. However, BMI is not significantly affected by the change in income inequality. Second, when measuring income distribution by the Gini coefficient, we find an inverted U-shaped relationship between the Gini coefficient and all health measures except for BMI, which means that income inequality has a positive effect on health status when it is low, but once a certain threshold is exceeded, it has a negative effect on individuals’ health. In addition, the results show that the widening of the income gap amplifies the effect of personal income on health, which implies that the increasing level of income inequality has caused the health conditions of poor people to deteriorate more over the study people than their rich counterparts. We also use different measures of income distribution, the Theil index and P90/P10, and the findings are mostly consistent. Lastly, we attempt to analyse the possible channels of the effects by analysing the accessibility of healthcare facilities and public infrastructures. The results indicate that income distribution affects individuals’ health by affecting the number of healthcare facilities and public infrastructures. The expansion of income inequality also increases the possibility and frequency of people engaging in hazardous health behaviours such as smoking and alcohol consumption.

In conclusion, China’s current income inequality is significantly higher than the international warning line, and this study shows that it is taking a toll on people’s health. To alleviate this problem, the Chinese government should use all means to reduce income inequality and speed up investment in healthcare facilities and public infrastructures, especially in rural areas.
